# Biological Functions and Applications of Exosomes from Periodontal Ligament Stem Cells

**DOI:** 10.3390/proteomes14030048

**Published:** 2026-09-10

**Authors:** Omer Tarik Ozdemir, Hideki Sugii, Bara Mardini, Hidefumi Maeda

**Affiliations:** 1 Department of Endodontology and Operative Dentistry, Faculty of Dental Science, Kyushu University, Fukuoka 812-8582, Japan; omertarik.ozdemir@ogr.iu.edu.tr (O.T.O.); bara.mardini@dent.kyushu-u.ac.jp (B.M.);; 2 Department of Pedodontics, Faculty of Dentistry, Istanbul University, 34134 Istanbul, Turkey; 3 Department of Endodontology, Kyushu University Hospital, 3-1-1 Maidashi, Higashi-ku, Fukuoka 812-8582, Japan

**Keywords:** exosome, periodontal ligament stem cell, periodontal ligament stem cell-derived exosome

## Abstract

Periodontal regeneration requires the reconstruction of root cementum, periodontal ligament, and alveolar bone, which are frequently disrupted by periodontitis, trauma, and other inflammatory conditions. Although periodontal ligament stem cells (PDLSCs) have been considered promising for periodontal tissue engineering because of their multipotency, self-renewal capacity, and immunomodulatory activity, direct cell-based therapy still faces various limitations, including donor variability, culture-related changes, delivery difficulties, and safety concerns. Exosomes and small extracellular vesicles derived from PDLSCs have therefore attracted attention as potential cell-free mediators of PDLSC paracrine activity. PDLSC-derived exosomes carry bioactive molecules, including proteins, lipids, messenger RNAs, and microRNAs, and can influence recipient cell behavior in periodontal and bone regenerative environments. In vitro and preclinical animal studies indicate that PDLSC-derived exosomes are involved in osteogenesis, periodontal attachment repair, cementogenesis, angiogenesis, mechanotransduction, cell proliferation/survival, and immune regulation. Their biological effects are closely related to the condition of their parental PDLSCs, their cargo, and the exosome isolation and characterization methods used. In this review, we summarize recent findings on the isolation strategies used, biological functions of, and therapeutic potential of PDLSC-derived exosomes, with particular focus on protein cargo, proteomic signatures, proteoform-related interpretation, and microRNA-mediated regulation. We also discuss current challenges in exosome isolation, characterization, delivery, stability, and reporting standards for future periodontal regenerative applications. The findings discussed throughout are preclinical, and their translation to clinical settings remains to be explored.

## 1. Introduction

The periodontal ligament (PDL) is specialized connective tissue that links root cementum to alveolar bone and supports tooth function under continuous mechanical loading. Periodontal inflammation or trauma can disrupt this organized attachment and lead to the progressive loss of cementum, periodontal ligament fibers, and supporting bone. True periodontal regeneration remains difficult because repair requires the coordinated reconstruction of multiple tissues rather than the simple filling of bone or relief of inflammation [1].

Periodontal ligament stem cells (PDLSCs) are resident clonogenic and multipotent progenitor populations within the periodontal ligament. Their tissue origin makes them especially relevant to periodontal regeneration, as they are directly associated with the formation and maintenance of cementum, periodontal ligament fibers, and alveolar bone [2]. Studies have supported the relevance of PDLSCs to periodontal regeneration while also emphasizing that this population is heterogeneous and influenced by donor-related and culture-related variables [3]. Transplantation studies in preclinical periodontal defect models further support the regenerative potential of PDLSCs, including cementum/PDL-like tissue formation and periodontal repair in large-animal settings [4,5]. Comparative proteomic studies also indicate that stem cells of dental origin have distinct molecular profiles, supporting the idea that PDLSCs should be interpreted as a site-specific regenerative cell population rather than as a source of generic mesenchymal stem cells [6,7]. The clinical translation of cell-based PDLSC therapy remains limited to a small number of controlled trials, ranging from autologous PDLSC sheets combined with bone grafting material in periodontal intrabony defects [8] to the direct application of an autologous PDLSC niche in periodontal osseous defects [9].

Despite this regenerative potential, direct cell transplantation has practical limitations, including donor variability, culture-related changes, delivery challenges, and regulatory complexity. This has shifted attention toward paracrine mechanisms, including conditioned medium, secretome, and extracellular vesicle-based approaches. In research on PDLSCs, conditioned medium has been linked to periodontal regenerative effects, supporting the concept that part of PDLSCs’ activity is mediated through secreted factors rather than cell replacement alone [10].

PDLSC-derived exosomes (PDLSC-Exo) or small extracellular vesicles have been investigated in various regeneration-related contexts. They reportedly exert functions in osteogenic differentiation, angiogenic responses, inflammatory modulation, macrophage-related immune regulation, and mechanically responsive remodeling, among others. In vivo studies have also linked PDLSC-Exo delivery to periodontal or bone regenerative outcomes, including alveolar bone defect repair and periodontitis-associated bone preservation [11,12,13,14]. Together, these findings point to therapeutic interest in PDLSC-Exo while also showing that their biological activity depends on donor cell state, conditioning, recipient cell context, and delivery strategy [15].

Two recent reviews by Ahmad et al. addressed related territories: one was on isolation methods across dental stem cell-derived exosomes [16], and the other conducted a literature mapping of PDLSC-Exo mechanisms in periodontal tissue regeneration [15]. The present review differs in scope and analytical framework: beyond periodontal regeneration, it also classifies reported proteins and microRNA (miRNA) into vesicle identity markers, functional cargo, and recipient cell pathway readouts, distinguishing confirmed cargo from downstream effects throughout. Thus, this review summarizes recent findings on the isolation strategies used, biological functions of, and therapeutic potential of PDLSC-Exo through protein cargo, proteomic signatures, proteoform-related interpretation, and microRNA-mediated regulation.

## 2. Periodontal Ligament Stem Cells

PDLSCs are resident clonogenic and multipotent progenitor populations within the periodontal ligament. Their importance in regenerative dentistry comes from their site-specific identity. Unlike more general mesenchymal stem cell sources, PDLSCs are directly associated with the formation, maintenance, and repair of cementum, periodontal ligament fibers, and alveolar bone. This tissue origin makes PDLSCs one of the most functionally relevant dental stem cell populations for periodontal regeneration [2,3].

The regenerative capacity of PDLSCs is supported by their potential to undergo osteogenic, cementogenic, adipogenic, and fibroblastic differentiation under appropriate conditions. Transplantation-based evidence further indicates that PDLSCs can contribute to cementum/PDL-like tissue formation in vivo, linking them directly to periodontal reconstruction rather than only nonspecific bone repair [2]. Comparative proteomic analyses of stem cells of dental origin also support this tissue-related profile, showing that PDLSCs possess cellular and secretory signatures associated with differentiation, proliferation, extracellular matrix organization, inflammation, and immune response [7].

Within their native niche, PDLSCs are continuously exposed to mechanical loading, microbial and inflammatory mediators, vascular signals, and extracellular matrix cues. These stimuli shape their osteogenic, immunomodulatory, angiogenic, and reparative behavior. PDLSCs also participate in local immune–stromal regulation through soluble mediators and cell contact-related mechanisms, including prostaglandin E2-associated immunomodulatory signaling [17,18]. This context-dependent behavior helps explain why PDLSC-Exo cargo and function may vary according to the parental cell state [15].

## 3. Exosome Biology and PDLSC-Derived Exosomes

Extracellular vesicles (EVs) are membrane-bound particles released by cells into the extracellular environment. They are commonly described as exosomes, microvesicles, or apoptotic bodies according to their size, origin, and biogenesis [19]. Exosomes are generally small EVs of approximately 30–150 nm in diameter and are generated through the endosomal pathway, whereas microvesicles bud directly from the plasma membrane and apoptotic bodies originate from fragmented apoptotic cells [19,20]. However, these categories are not always fully separable in experimental preparations because their size ranges, densities, and surface markers can overlap [21].

Exosomes carry selected molecular cargo, including proteins, lipids, metabolites, messenger RNAs (mRNAs), miRNAs, and other non-coding RNAs. By transferring these molecules to recipient cells, they contribute to intercellular communication in physiological and pathological processes. This cargo-mediated signaling is one reason why EVs are increasingly studied as paracrine mediators in regenerative medicine and tissue repair [22,23].

Exosome-enriched preparations are commonly characterized by the presence of tetraspanins such as CD9, CD63, and CD81, together with endosomal or endosomal sorting complex required for transport (ESCRT)-associated proteins such as TSG101 and ALIX. These markers support vesicle enrichment, but they are not specific to a single EV subtype. Current EV guidance therefore recommends reporting complementary characterization data, including particle size and concentration, morphology, positive markers, and negative controls for cellular contamination [21,24].

### 3.1. Exosome Biology

Exosome biogenesis is regulated by intracellular sorting pathways. In many settings, ESCRT promotes inward budding within multivesicular bodies (MVBs), leading to intraluminal vesicle formation. These vesicles are released as exosomes when MVBs fuse with the plasma membrane. Alternatively, ESCRT-independent routes have also been described, where lipid rafts and tetraspanins contribute to cargo sorting and vesicle budding [25,26]. Pathways can vary by cell type. Ceramide accumulation in the endosomal membrane can promote vesicle budding through an ESCRT-independent mechanism. These processes generate multivesicular bodies filled with small intraluminal vesicles. When multivesicular bodies fuse with the plasma membrane, the vesicles are released as exosomes carrying selected subsets of cellular cargo. This biogenesis route contributes to characteristic features, including an enrichment in endosomal membrane proteins and a specific lipid composition, and helps distinguish exosomes from other extracellular vesicle classes [27].

### 3.2. PDLSC-Derived Exosomes

PDLSC-derived exosomes represent a vesicular component of PDLSC paracrine signaling. Their cargo reflects the biological state of the parental PDLSCs and may include proteins, lipids, and regulatory RNAs associated with periodontal repair, inflammatory responses, and tissue remodeling. This concept is consistent with evidence that PDLSC-conditioned medium and secretome components can influence periodontal regeneration-related processes [10,15,28].

Minimal Information for Studies of Extracellular Vesicles 2023 (MISEV2023) discourages using the term “exosome” unless subcellular origin is demonstrated, recommending “small EV” instead [21]. None of the studies reviewed here meet this standard, yet nearly all describe their vesicles as exosomes. In this review, the term “PDLSC-Exo” is used in line with the terminology of the cited literature and is the only abbreviation used for these vesicles. Where a study reported extracellular vesicles instead, we use that full term rather than a separate abbreviation. The following sections therefore discuss the biology of PDLSC-Exo through their reported functions, protein cargo, proteome-level signatures, and miRNA-mediated regulatory mechanisms.

## 4. Isolation of Exosomes from PDLSCs

PDLSC-Exo are usually obtained from conditioned culture medium or the serum of established cell lines [12], commercially sourced PDLSCs [29], and extracted molar and premolars [13,14,30], so preanalytical control starts at the culture stage. Serum supplementation can introduce bovine vesicles that confound both quantification and cargo profiling, and many PDLSC studies therefore use extracellular vesicle-depleted fetal bovine serum (FBS) prepared by prolonged ultracentrifugation, or they condition cells in a low-serum setting for a limited time window [11,31]. A direct PDLSC comparison of FBS against human platelet lysate has not yet been reported. In other MSC types, platelet lysate has increased vesicle yield and enriched angiogenic cargo [32]. Culture conditions also influence vesicle secretion and cargo. This extends beyond the proliferation rate: donor age, tooth health status, and passage number are known to shape vesicle yield and cargo composition [7]. Inflammatory priming and osteogenic induction are further variables that should be considered when comparing baseline and preconditioned PDLSC preparations [33].

**Figure 1:**
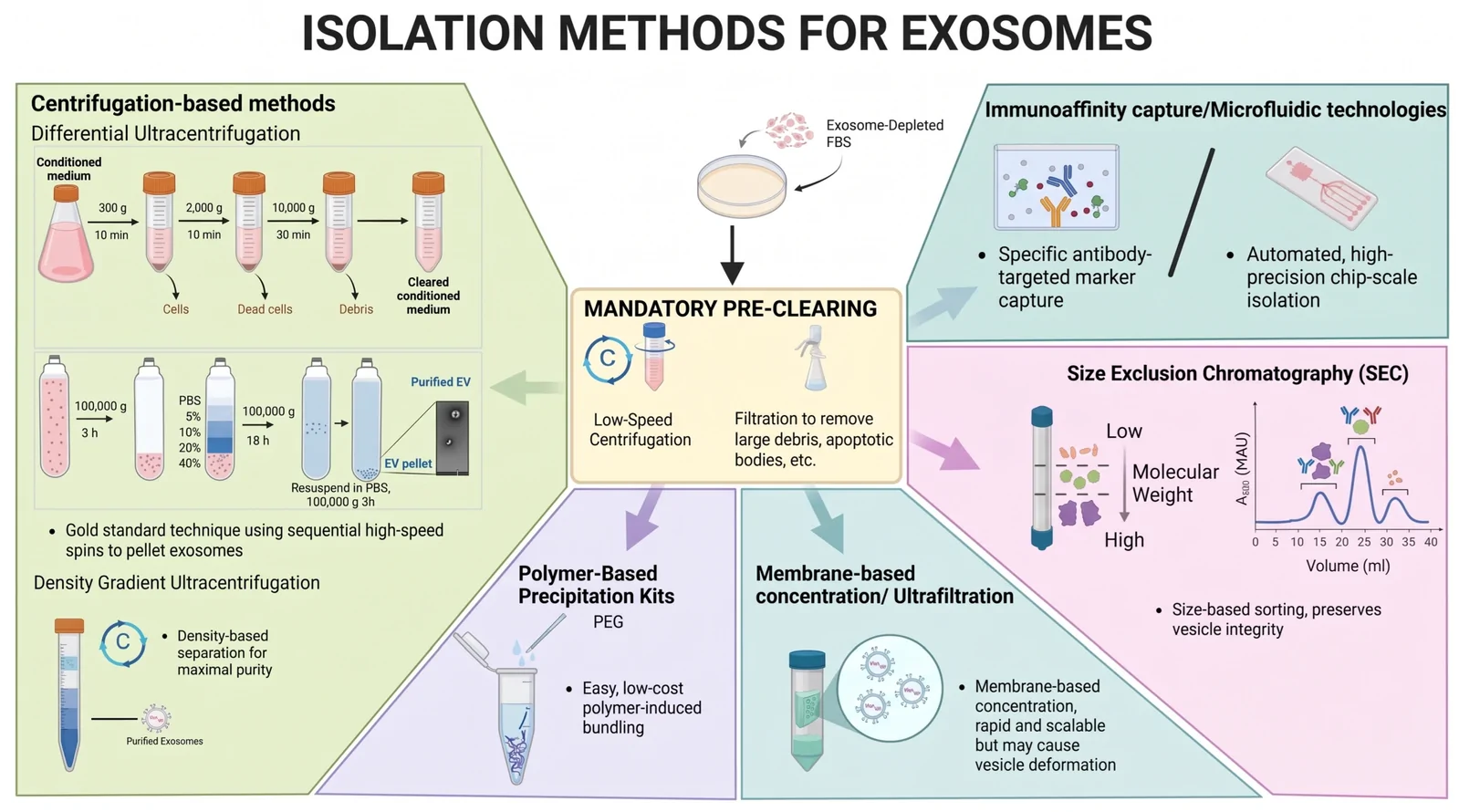
Common strategies for isolating PDLSC-derived exosomes or small extracellular vesicles.

After conditioning, the medium is collected under sterile conditions and pre-cleared to remove intact cells and large debris. Most workflows use sequential low-speed centrifugation followed by filtration through a small-pore membrane to reduce the carryover of apoptotic bodies and large microvesicles. This step is particularly important for proteomic or RNA profiling because residual debris can dominate downstream signals [34]. Common strategies for isolating PDLSC-derived exosomes or small extracellular vesicles are schematically summarized in Figure 1.

### 4.1. Ultracentrifugation

Differential ultracentrifugation is the most frequently reported approach for enriching PDLSC-Exo from conditioned medium [35]. A typical workflow applies increasing centrifugal forces to remove remaining large particles and then applies high-speed ultracentrifugation to pellet small vesicles, followed by a washing step to reduce soluble protein carryover. This method is widely used because it is accessible and scales to moderate volumes, yet it can also increase vesicle aggregation and co-pellet nonvesicular protein complexes. Accordingly, interpretation is facilitated by the inclusion of negative controls and, when required, additional purification steps [34].

Density gradient ultracentrifugation is often used as a secondary purification step after differential ultracentrifugation when higher purity is required. Iodixanol or sucrose gradients separate particles by buoyant density, which can reduce contamination by soluble proteins and lipoproteins that co-sediment in simple pelleting workflows [36]. This approach is commonly preferred when downstream cargo profiling is planned because modest differences in purity can translate into large apparent differences in protein or miRNA content [37].

### 4.2. Size Exclusion Chromatography (SEC)

SEC separates vesicles from smaller soluble proteins based on hydrodynamic size and is frequently selected when vesicle integrity and functional activity must be preserved. In practice, chromatography is often combined with an upstream concentration step such as ultrafiltration because conditioned medium volumes can be large. The main limitation is that yield can be lower than in precipitation-based approaches, so SEC is typically chosen when purity and structural preservation are prioritized over maximizing recovery [38,39].

### 4.3. Ultrafiltration

Ultrafiltration concentrates vesicles using molecular weight cutoff membranes and can reduce processing time compared with repeated ultracentrifugation. It is most often used as a concentration step before SEC or density gradient separation rather than as a stand-alone purification method. In ultrafiltration, vesicle loss can occur due to membrane adhesion and clogging, and shear forces may affect vesicle integrity, depending on the applied pressure and flow conditions [40]. For these reasons, ultrafiltration has often been paired with SEC to separate vesicles from soluble proteins after concentration [41].

### 4.4. Polymer-Based Precipitation

Polymer-mediated precipitation, commonly based on polyethylene glycol, is widely used because it is simple and does not require an ultracentrifuge. Commercial kits built on this principle can rapidly enrich vesicles from conditioned medium and are often selected for exploratory studies or high-throughput workflows. A major limitation is lower purity due to the co-precipitation of proteins and other macromolecular assemblies, which can complicate proteomic analyses and introduce artifacts in uptake or functional assays if residual polymer is retained [34].

### 4.5. Immunoaffinity-Based Capture and Microfluidic Platforms

Affinity-based isolation uses surface markers such as CD9, CD63, and CD81 to capture vesicles, which can increase specificity when a targeted vesicle subset is required. The trade-off is that the captured fraction may not represent the full PDLSC-derived vesicle population and the yield can be limited, making this approach more suitable for biomarker studies than for bulk functional applications [42]. Microfluidic platforms have been developed to integrate isolation with analysis using small sample volumes, yet standardization and inter-platform comparability remain active issues. Accordingly, these methods are commonly reported alongside established isolation approaches rather than acting as direct replacements for them [20,42,43].

### 4.6. Strengths, Limitations, and Rationale for Combined Isolation Approaches

Differential ultracentrifugation offers broad accessibility and high vesicle yield, yet high-speed centrifugal forces have been associated with vesicle aggregation, membrane damage, and soluble protein co-pelleting that can compromise downstream cargo profiling [34,44]. SEC preserves vesicle integrity and reduces lipoprotein contamination more effectively than pelleting-based workflows, making it better suited to proteomic applications, albeit at lower recovery [45]. Ultrafiltration and polymer-based precipitation are operationally simpler but yield preparations of insufficient purity for unbiased cargo profiling, while immunoaffinity-based methods achieve the highest specificity at the cost of yield and representative population coverage [34,40].

These trade-offs provide the rationale for combining isolation methods rather than relying on any single approach. A systematic analysis of EV methods in publications found that approximately 60% of isolations used combined methods, which consistently outperformed single methods for both purity and yield regardless of starting material type [46]. Low-speed centrifugation paired with SEC provides the highest EV purity, while SEC followed by ultracentrifugation is particularly suited when proteomic profiling is planned, and ultrafiltration paired with SEC is commonly selected for conditioned medium-based workflows [40,41,46,47]. For PDLSC-Exo in particular, where parental cell conditioning and donor-related variability already shape vesicle cargo composition, the isolation strategy should be aligned with the intended downstream application [16].

## 5. Function of Exosomes from PDLSCs

**Figure 2:**
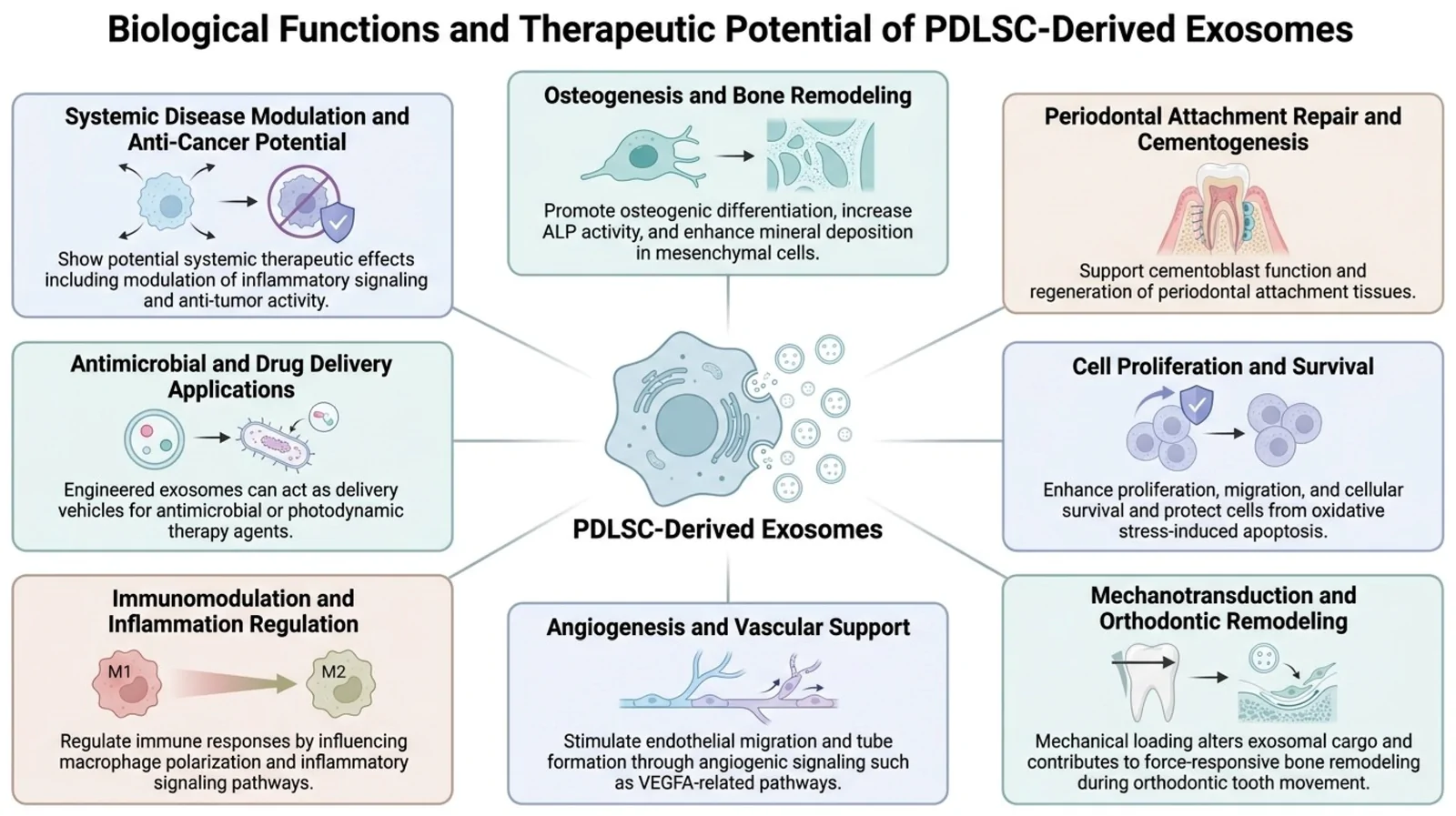
Biological functions and therapeutic potential of PDLSC-derived exosomes.

In vitro and in vivo models have linked PDLSC-Exo to diverse biological functions. To present these findings more clearly, the reported effects were grouped into eight functional domains, as summarized in Figure 2.

### 5.1. Osteogenesis and Bone Remodeling

Osteogenesis is one of the most consistently reported functions of PDLSC-Exo. In an osteogenic model, increased osteoblast-lineage activity, alkaline phosphatase activity, mineral deposition, and osteogenic marker expression were also observed [11]. Donor cell conditioning may influence these effects, as exosomes derived from osteogenically induced PDLSCs showed stronger osteogenicity than those from undifferentiated PDLSCs [11]. A complementary line of evidence has shown that PDLSC-derived extracellular vesicles can be internalized by recipient bone marrow mesenchymal stem cells (BMMSCs) and enhance osteogenic differentiation through EV secretion- and uptake-related mechanisms [30]. In vivo bone defect studies further support the contribution of PDLSC-Exo to local bone repair and osteoblast-like cell differentiation [12].

### 5.2. Periodontal Attachment Repair and Cementogenesis

Beyond general bone formation, PDLSC-Exo are relevant to periodontal attachment repair because their parental cells originate from the periodontal ligament niche. In periodontitis-related models, local PDLSC-Exo delivery has been associated with reduced alveolar bone destruction and improved periodontal repair-related outcomes [48]. Cementoblast-focused studies also suggest that PDLSC-Exo can enhance biological activity in cells directly related to cementum formation and periodontal attachment repair [49].

### 5.3. Cell Proliferation, Migration, and Survival

PDLSC-Exo also regulate basic cellular behaviors that support tissue repair. Increased proliferation and migration have been reported in recipient osteoblast-like or mesenchymal cells after PDLSC-Exo exposure [48]. Under oxidative stress conditions, PDLSC-Exo reduced apoptosis and supported cell survival while also activating survival-related signaling pathways [50].

### 5.4. Mechanotransduction and Orthodontic Remodeling

PDLSC-Exo function is closely linked to the mechanical environment of the periodontal ligament. Mechanical loading has been reported to alter exosomal cargo and recipient cell responses during force-related remodeling. In this context, mechanically stimulated PDLSC-Exo showed changes in functional cargo associated with vesicle uptake, ERK signaling, and bone remodeling responses [51]. In an orthodontic tooth movement model, delivery on the tension side was associated with improved trabecular bone parameters and increased osteogenic marker expression [52]. These studies connect function with force-responsive periodontal remodeling.

### 5.5. Angiogenesis and Vascular Support

Angiogenesis-related effects form another functional domain of PDLSC-Exo, particularly in contexts of periodontal inflammation where vascular support and tissue repair are closely linked. In endothelial cell models, PDLSC-Exo increased tube formation and angiogenic marker expression; blocking vesicle release reduced these angiogenic responses. The same study linked this effect to exosomal VEGFA transfer and miR-17-5p-related regulation, providing a functional connection between PDLSC-Exo and endothelial behavior in an inflammatory periodontal microenvironment [13].

### 5.6. Immunomodulation and Inflammation Regulation

The immunological effects of PDLSC-Exo are strongly context-dependent. In adaptive immune models, PDLSC-Exo modulate Th17/Treg balance through miRNA-associated pathways such as miR-155-5p/SIRT1 and miR-205-5p/XBP1 [29,53]. In macrophage models, LPS-primed PDLSC small extracellular vesicles (sEVs) promoted inflammatory macrophage responses through TLR2/TLR4/NF-κB-related signaling [54]. In vivo periodontitis studies also reported that inflammatory PDLSC-Exo could aggravate periodontal inflammation by promoting M1 macrophage polarization [55]. Together, these results point to a context-dependent role for PDLSC-Exo in immunomodulation, especially when donor cells are exposed to inflammatory stimulation.

### 5.7. Antimicrobial and Drug Delivery Applications

A small number of studies used PDLSC-Exo as delivery vehicles rather than as native regenerative mediators. Emodin-loaded PDLSC-Exo reduced bacterial viability and biofilm formation against cariogenic bacteria under antimicrobial photodynamic therapy [56], and kojic acid-loaded PDLSC-Exo reduced Enterococcus faecalis biofilm load and virulence gene expression under antimicrobial sonodynamic therapy [57]. In both cases, the functional contribution reflects exosome-enabled payload delivery.

### 5.8. Systemic Disease Modulation and Anti-Cancer Potential

PDLSC-Exo were also explored in early preclinical models beyond local periodontal repair. In neuroinflammatory models, PDLSC-derived secretomes or exosome-enriched fractions regulated inflammasome-related inflammatory responses and reduced downstream cytokine release [58,59]. In metabolic stress models, PDLSC-Exo were reported to attenuate high-glucose-associated senescence through stress adaptation pathways [60]. More recently, PDLSC-Exo were investigated in cancer-related models, where they reduced cancer cell growth, colony formation, and motility and enhanced chemosensitivity in combination with oxaliplatin [61]. Non-coding RNA profiling has also suggested links between cargo and tumor-related signaling pathways [62]. No clinical study has yet evaluated PDLSC-Exo in these systemic settings, so the results demonstrate biological capacity, not therapeutic readiness.

## 6. Proteins Inside Exosomes from PDLSCs

Exosomal proteins are important for two practical reasons. First, they are used to characterize exosome- or small extracellular vesicle-enriched preparations. Most isolation workflows cannot confirm endosomal biogenesis directly and may co-isolate other small extracellular particles. Current community guidance therefore recommends reporting a marker panel with appropriate negative controls and interpreting exosome claims cautiously when subtypes are incompletely separated [21]. Second, exosomal proteins can act as bioactive cargo. They can influence recipient cell behavior through receptor interactions, membrane fusion or endocytosis, and the delivery of signaling components [51].

Common exosome-associated proteins include the tetraspanins CD9, CD63, and CD81, together with endosomal or ESCRT-associated proteins such as TSG101 and ALIX. These markers support an exosome/sEV-enriched preparation, but they are not exclusive to a single vesicle subtype. Negative markers, such as calnexin, are equally important because they help assess cellular or endoplasmic reticulum contamination. For example, an osteogenesis-focused PDLSC-Exo study has combined positive markers such as CD63 and TSG101 with calnexin depletion to support vesicle enrichment and exclude cellular carryover [11]. Therefore, PDLSC-Exo characterization should rely on marker panels and appropriate controls, rather than positivity for a single protein [21].

Many reported PDLSC-Exo cargo proteins are inferred from functional or blocking experiments rather than confirmed by direct proteomic detection within purified vesicles. VEGFA and ANXA3 illustrate this range. VEGFA is a major angiogenic factor that regulates endothelial cell behavior and vascular network formation, processes closely linked to periodontal tissue repair. Zhang et al. detected VEGFA within isolated PDLSC-Exo by targeted Western blot. Its causal role in the angiogenic effect was not isolated from total exosome secretion [13]. ANXA3 belongs to the annexin family, a group of calcium-dependent phospholipid-binding proteins frequently detected in exosome-enriched preparations [51]. Huang et al. identified ANXA3 through the proteomic profiling and Western blot of mechanically stimulated PDLSC-Exo, then showed that ANXA3 facilitated exosome uptake and activated ERK signaling to promote osteoclast-related remodeling [51]. Neither protein’s intraluminal localization has been confirmed by protease protection assay.

Many proteins reported in PDLSC-Exo studies are better interpreted as recipient cell responses or pathway readouts. Osteogenic proteins such as RUNX2, ALP, OCN, OPN, BMP2, and BMP4 usually reflect differentiation and mineralization after PDLSC-Exo treatment, rather than confirmed protein cargo within the vesicle fraction [11,12,28]. Immune and inflammatory proteins such as TLR2, NF-κB, NLRP3, caspase-1, IL-1β, TNF-α, IL-10, SIRT1, XBP1, and FoxO1 similarly reflect macrophage polarization, pyroptosis, Th17/Treg balance, or the modulation of stress-related pathways after vesicle exposure [19,29,53,54,63]. A similar cytokine and apoptotic profile has been reported for engineered, MSC-derived exosomes in other inflammatory disease models [64]. Secretome-associated proteins, including FN1, COL1A1, COL1A2, THBS1, and keratins, further support the extracellular matrix and adhesion-related character of PDLSC paracrine signaling but should not be described as exosomal cargo unless purified EV proteomics confirms their enrichment [28]. Table 1 summarizes representative proteins and protein-related signals reported in PDLSC-Exo studies, including molecules used for exosome/sEV characterization and those associated with the biological effects of PDLSC-Exo on recipient cells.

## 7. MicroRNAs Inside Exosomes from PDLSCs

Exosomal miRNAs are regulatory cargo molecules that connect the biological state of PDLSCs with the behavior of recipient cells. By controlling post-transcriptional gene networks, PDLSC-Exo miRNAs can influence osteogenic commitment, inflammatory signaling, endothelial responses, and stress adaptation. Their function should therefore be interpreted at the pathway level rather than as isolated effects of single molecules [15,21].

Bone remodeling is the most consistently represented functional domain of PDLSC-Exo miRNAs. Osteogenic induction changes the miRNA cargo of PDLSC-Exo supports osteogenic activity in recipient mesenchymal cells [11]. Specific miRNA axes also connect PDLSC-Exo with periodontal bone repair, including miR-31-5p/eNOS in alveolar bone regeneration [48], miR-200b/c–Smurf1–BMP/Smad signaling in inflammatory bone loss [66], and miR-141-3p-KEAP1/NRF2 in high-glucose-associated senescence and stress adaptation [60]. These mechanisms position PDLSC-Exo miRNAs as regulators of mineralized tissue repair rather than passive cargo markers.

PDLSC-Exo miRNAs also participate in vascular and mechanical adaptation. In inflammatory periodontal environments, the miR-17-5p-mediated control of VEGFA contributes to endothelial behavior and angiogenic responses [13]. Mechanical stimulation further changes the miRNA profile of PDLSC-Exo and links vesicular cargo to force-responsive remodeling pathways [67,68]. These findings place vascular regulation and mechanical adaptation alongside osteogenesis as recurring functional themes in PDLSC-Exo miRNA biology.

The immunomodulatory effects of PDLSC-Exo miRNAs are strongly context-dependent. Some miRNA axes attenuate inflammation and restore immune balance, such as miR-205-5p/XBP1 and miR-155-5p/SIRT1 in the Th17/Treg axis [53]. Others can amplify inflammatory responses under disease or mechanically stressed conditions, including miR-143-3p–PI3K/AKT/NF-κB, miR-433-3p–TLR2/TLR4/NF-κB, and miR-9-5p–SIRT1/NF-κB signaling [54,55,69]. Thus, PDLSC-Exo miRNAs should not be described as uniformly anti-inflammatory or pro-regenerative; their biological direction depends on donor cell conditioning, recipient cell type, and periodontal microenvironment.

Not all periodontal vesicle-related RNA mechanisms originate directly from PDLSCs or involve miRNAs only. PDL fibroblast-derived exosomes carrying miR-34c-5p can suppress PDLSC osteogenic differentiation through the SATB2/ERK pathway [70], whereas high-glucose-induced miR-129-3p accumulation in PDLSC links impaired exocytosis to ER calcium imbalance and RUNX2 degradation [71]. In addition, osteogenically induced PDLSC-Exo may carry non-miRNA regulatory cargo, such as circ_0000722, which has been associated with TRAF6/AKT/NF-κB-related osteoclastogenic signaling [72]. Therefore, we separated functionally defined PDLSC-derived exosomal miRNA axes from related periodontal vesicle mechanisms and profiling-only candidates. The validated PDLSC-derived exosomal/sEV miRNA axes are summarized in Table 2.

## 8. Proteomes Inside Exosomes from PDLSCs

The proteome of PDLSC-Exo is inherently associated with the biological state of the parental PDLSCs. Vesicular cargo is selected from a cell population already specialized for periodontal remodeling, matrix interaction, mechanical adaptation, and inflammatory response. Therefore, PDLSC-Exo cargo should be interpreted as a condition-dependent extension of the PDLSC proteome, not as a fixed molecular signature.

The parental PDLSC proteome provides the first biological background for this interpretation. PDLSCs contain protein networks related to cytoskeletal remodeling, metabolic activity, redox control, and osteogenic commitment, all of which are relevant to periodontal repair. These donor cell programs help explain why PDLSCs are repeatedly associated with migration, matrix remodeling, osteogenesis, and adaptation to inflammatory or mechanical stress [6,73].

The secretome represents the next layer between the intracellular proteome and exosome cargo. PDLSC-conditioned medium contains an extracellular protein environment enriched in matrix-related, angiogenic, migratory, inflammatory, and osteogenic components. Although conditioned medium is not exosome-specific, it defines the broader paracrine protein landscape from which exosome-enriched fractions are derived [7,28].

Direct PDLSC-Exo profiling supports this condition-dependent view. Mechanical loading changes exosomal protein cargo, with ANXA3 acting as a functional example linked to vesicle uptake, ERK signaling, and osteoclast differentiation [51]. Osteogenic induction also reshapes exosomal regulatory cargo, including miRNAs, circRNAs, and lncRNAs associated with osteogenic and cytoskeletal pathways [74]. Thus, PDLSC-Exo function is likely driven by coordinated protein and RNA cargo programs rather than by a single molecule.

Proteomic methods for vesicle characterization have shifted over time from gel-based protein separation toward mass spectrometry-driven discovery [75]. Early workflows separated proteins by two-dimensional electrophoresis before identifying individual spots through peptide mass fingerprinting, an approach reflected in the early proteomic profiling of the PDLSC proteome itself [6]. Modern liquid chromatography–tandem mass spectrometry instead fragments peptides directly, using either data-dependent acquisition (DDA) or data-independent acquisition (DIA) [76]. However, targeted antibody-based detection can only confirm proteins selected in advance and offers no discovery capacity.

A complete proteome map of PDLSC-Exo is still lacking. Current evidence is stronger for donor cell proteomes, conditioned medium proteomics, and RNA cargo profiling than for unbiased exosomal protein catalogs. This partly reflects the fact that most of the studies cited in Table 1 rely on the targeted detection of a small, predetermined panel of proteins, such as CD9, CD63, CD81, and TSG101, rather than untargeted proteomic profiling that identifies proteins without prior selection [11,12,14,29]. A targeted panel can confirm whether these specific proteins are present but cannot reveal what else the vesicle preparation may contain. Among the small number of studies that did use untargeted profiling, only two report a false discovery rate threshold for their protein identifications, both using a 1% cutoff. However, they relied on different search engines and quantification strategies, which limits direct comparison between them [28,61]. For the remaining literature, the proportion of reported proteins that could be false identifications cannot be assessed. Most available proteomic workflows infer protein or protein group abundance from peptide-level data and rarely resolve intact proteoforms. For this reason, PDLSC-Exo proteomic findings should be described in terms of protein or proteoform abundance, rather than unsupported claims of protein expression or regulation [77].

Several factors account for this gap. Primary PDLSC cultures carry substantial inter-patient variability that immortalized cell lines do not. Donor sex shapes secretory yield and the balance of anti-inflammatory mediators in other MSC types, yet it remains unexamined for PDLSC-Exo [78]. Culture medium adds a further layer of variability. Fetal bovine serum remains the default choice, but even EV-depleted FBS retains residual bovine vesicles and reduces the medium’s capacity to support cell growth [79]. Protein cargo profiles also vary with the isolation method used, limiting direct cross-study comparison [80]. Even within a single isolation approach, protein detection across biological replicates shows substantial qualitative variability, independent of actual protein abundance [81]. The available PDLSC-Exo studies show the same limitation directly. The most rigorous untargeted study reported here analyzed four experimental groups using a single biological sample per group, relying on technical replicates to assess measurement consistency rather than donor-to-donor variability [61]. Certain studies do not even report basic details such as antibody dilution or clone [14,54]. A comparable validation gap affects the antibodies used throughout this literature. Standard guidance recommends confirming specificity against a knockdown or knockout control [82], yet none of the studies reviewed here report this step, so a positive Western blot signal indicates that the antibody reacted with something of the expected molecular weight rather than confirming the target protein’s identity.

The Human Proteome Organization’s Proteomics Standards Initiative recommends reporting an explicit false discovery rate threshold as a minimum standard for proteomic identifications [80]. Addressing this gap will require proteomics performed directly on purified vesicle fractions rather than conditioned medium, using untargeted approaches that meet this reporting standard [76]. Harmonized isolation workflows and the consistent co-reporting of donor characteristics, passage number, and culture parameters would further enable cross-study comparison [83]. Only one study reviewed here deposited its raw proteomic data to a public repository, ProteomeXchange/JPOST [61]. The deposition of PDLSC-Exo proteomic datasets to open access repositories such as ExoCarta and Vesiclepedia would provide a shared reference framework currently absent for this cell type [84,85].

## 9. Future Perspectives and Improvement

PDLSC-derived exosomes and small extracellular vesicles have been linked to osteogenic remodeling, angiogenic support, and immune regulation in periodontal and related regenerative models. The next priority is to improve the standardization of what is isolated, tested, and claimed. Current studies still vary in separation workflows, characterization depth, dose normalization, and functional controls. Frameworks such as Transparent Reporting and Centralizing Knowledge in Extracellular Vesicle Research (EV-TRACK) and MISEV2023 provide practical guidance for transparent reporting and cross-study comparability and should be treated as core requirements in future PDLSC-Exo studies [21,86].

A second priority is cargo-level definition. Many functional studies report downstream biological effects, but fewer studies define vesicle cargo with sufficient proteomic depth. Future work should combine exosome characterization with protein cargo profiling, proteoform-aware interpretation, and the public deposition of raw omics data where possible. This would make it easier to distinguish vesicle markers, functional cargo, and recipient cell pathway readouts [87]. A third priority is scalable and clinically compatible manufacturing. Periodontal applications may require repeated local delivery, consistent batch production, defined critical quality attributes, and mechanism-aligned potency assays. The International Society for Extracellular Vesicles (ISEV) position paper on EV therapeutics provides a useful framework for quality, safety, and clinical trial readiness in EV-based products [88].

Delivery and stability also require more systematic evaluation. Periodontal tissues are vascularized and mechanically active, so freely delivered vesicles may be cleared rapidly and may benefit from biomaterial-based retention or controlled-release systems [14]. Storage conditions should also be treated as part of a product’s definition because freeze–thaw cycles, buffers, and preservation strategies can change EV integrity and bioactivity, as highlighted by recent systematic evidence on storage and freezing protocols [89]. Practical stabilizing buffer strategies have also been reported, supporting the idea that formulation is a controllable variable rather than a logistical afterthought [90]. Beyond stabilization, combining vesicles with engineered nanoparticles may further enhance therapeutic potential. The broader mesenchymal stem cell literature has reported increased anti-inflammatory activity when conditioned medium is paired with engineered nanoparticles [91]; selenium nanoparticle-based strategies alone have suppressed PI3K/AKT/NF-κB signaling in inflamed mesenchymal stem cells [92]; and nanoparticle biocompatibility has been highlighted as a key translational consideration for regenerative applications more generally [93].

Finally, early clinical exploration in periodontology is becoming more plausible, although it is not yet PDLSC-specific. A registered clinical study is testing adipose-derived stem cell exosomes as an adjunct to scaling, and root planing in periodontitis suggests that EV-based periodontal applications are moving toward human feasibility testing [94]. For PDLSC-Exo, the most reasonable next step is carefully designed early-phase work focused on safety, standardized batch definition, local inflammatory biomarkers, and imaging-based bone- or attachment-related outcomes [21].

## Figures and Tables

**Table 1:** Representative markers, cargo molecules, and pathway-associated readouts reported in PDLSC-derived exosome/sEV studies.

Category	Representative Markers	Biological Significance in PDLSCs	Reference Number
Identity Markers			
Tetraspanins (identity)	CD9, CD63, CD81	Used as membrane-enriched identifiers to support exosome-enriched status	[12,14,54,61,65]
Endosomal (biogenesis)	TSG101, ALIX, syntenin, flotillins	Supports endosomal sorting and biogenesis via the ESCRT pathway	[13,21]
Purity (negative control)	Calnexin (CANX)	Endoplasmic reticulum marker, the absence of which confirms the exclusion of cellular debris	[11,21]
Recipient Cell Readouts			
Functional cargo	ANXA3, VEGFA	Bioactive mediators shaping recipient cell behavior (measured directly in the vesicle)	[13,51]
Intercellular signaling/downstream readouts	p-AKT, p-ERK1/2	Reflect recipient cell signaling activation after PDLSC-EV exposure	[14]
Bone formation and calcification	RUNX2, OSX/SP7, ALP, OCN, OPN, BMP2, BMP4	Reflect osteogenic differentiation, mineralization, and bone repair responses after PDLSC-Exo treatment	[11,12,28]
Immune and inflammation regulation	TLR2, TLR4, NF-κB, p65, NLRP3, GSDMD, caspase-1, IL-1β, TNF-α, IL-10, SIRT1, XBP1, FoxO1	Reflect inflammatory signaling, macrophage polarization, pyroptosis, and Th17/Treg-related modulation	[53,54]
Adhesion/ECM remodeling	FN1, COL1A1, COL1A2, THBS1	Associated with extracellular matrix organization, adhesion, migration, and periodontal repair	[28]
Adhesion/cytoskeleton	KRT1, KRT10, KRT5, actin/focal adhesion-associated proteins	Associated with cytoskeletal organization and cell interaction-related paracrine signaling	[6,28]

**Abbreviations:** ALIX, ALG-2-interacting protein X; ALP, alkaline phosphatase; ANXA3, annexin A3; BMP, bone morphogenetic protein; CANX, calnexin; CD, cluster of differentiation; COL1A1, collagen type I alpha 1 chain; COL1A2, collagen type I alpha 2 chain; ECM, extracellular matrix; ESCRT, endosomal sorting complex required for transport; FN1, fibronectin 1; GSDMD, gasdermin D; IL, interleukin; KRT, keratin; NF-κB, nuclear factor kappa B; NLRP3, NOD-, LRR-, and pyrin domain-containing protein 3; OCN, osteocalcin; OPN, osteopontin; OSX/SP7, osterix/specificity protein 7; RUNX2, runt-related transcription factor 2; THBS1, thrombospondin 1; TLR, Toll-like receptor; TNF-α, tumor necrosis factor alpha; TSG101, tumor susceptibility gene 101; VEGFA, vascular endothelial growth factor A; XBP1, X-box binding protein 1.

**Table 2:** Functional miRNA axes reported in PDLSC-derived exosomes or small extracellular vesicles.

Functional Domain	miRNA Axis	EV Source/Conditioning	Main Target or Pathway	Biological Implication	Reference
Bone remodeling/periodontal regeneration	miR-31-5p	PDLSC-Exo; normal vs. high-glucose conditions	eNOS	Modulates osteoclastogenesis and alveolar bone regeneration	[48]
Osteogenesis/inflammatory bone loss	miR-200b/c	Tension-stimulated PDLSC-Exo/miR-200b/c-functionalized exosomes	SMURF1–BMP/Smad	Enhances osteogenic signaling and bone repair	[66]
Stress adaptation/senescence	miR-141-3p	PDLSC-Exo under high-glucose conditions	KEAP1–NRF2	Attenuates high-glucose-associated senescence and oxidative stress	[60]
Angiogenesis	miR-17-5p	Inflamed PDLSC-Exo/inflammatory periodontal microenvironment	VEGFA	Regulates endothelial angiogenic activity	[13]
Adaptive immune balance	miR-155-5p	PDLSC-Exo; chronic periodontitis context	SIRT1/Th17–Treg axis	Modulates Th17/Treg balance	[53]
Adaptive immune balance/inflammation	miR-205-5p	PDLSC-Exo; chronic periodontitis context	XBP1/Th17–Treg axis	Attenuates inflammatory signaling and supports immune rebalance	[29]
Macrophage polarization	miR-143-3p	Inflammatory PDLSC-Exo	PI3Kγ–PI3K/AKT/NF-κB	Promotes M1 macrophage polarization and inflammatory amplification	[55]
Macrophage polarization	miR-433-3p	LPS-primed PDLSC-sEVs	TLR2/TLR4/NF-κB p65	Promotes M1 macrophage polarization	[54]

**Abbreviations:** AKT, protein kinase B; eNOS, endothelial nitric oxide synthase; KEAP1, Kelch-like ECH-associated protein 1; LPS, lipopolysaccharide; NF-κB, nuclear factor kappa B; NRF2, nuclear factor erythroid 2-related factor 2; PDLSC-Exo, periodontal ligament stem cell-derived exosome; PI3K, phosphoinositide 3-kinase; sEV, small extracellular vesicle; SIRT1, sirtuin 1; SMURF1, SMAD-specific E3 ubiquitin protein ligase 1; TLR, Toll-like receptor; VEGFA, vascular endothelial growth factor A; XBP1, X-box binding protein 1.

## Data Availability

The original contributions presented in this study are included in the article; further inquiries can be directed to the corresponding author.

## Abbreviations

The following abbreviations are used in this manuscript:

DSC	Dental stem cell
ESCRT	Endosomal sorting complex required for transport
EV	Extracellular vesicle
EV-TRACK	Transparent Reporting and Centralizing Knowledge in Extracellular Vesicle Research
FBS	Fetal bovine serum
ISEV	International Society for Extracellular Vesicles
MISEV2023	Minimal Information for Studies of Extracellular Vesicles 2023
miRNA	MicroRNA
mRNA	Messenger RNA
MVB	Multivesicular body
PDL	Periodontal ligament
PDLSC	Periodontal ligament stem cell
PDLSC-Exo	Periodontal ligament stem cell-derived exosome
SEC	Size exclusion chromatography
sEV	Small extracellular vesicle
